# Episodic and Ongoing Mechanisms Drive Plastid-Derived Nuclear DNA Evolution in Angiosperms

**DOI:** 10.1093/gbe/evaf194

**Published:** 2025-10-13

**Authors:** Juan Pablo Marczuk-Rojas, Ana D Maldonado, Lorenzo Carretero-Paulet

**Affiliations:** Department of Biology and Geology, University of Almería, Almería 04120, Spain; “Pabellón de Historia Natural-Centro de Investigación de Colecciones Científicas de la Universidad de Almería” (PHN-CECOUAL), University of Almería, Almería 04120, Spain; Department of Mathematics and Center for the Development and Transfer of Mathematical Research to Industry (CDTIME), University of Almería, Almería 04120, Spain; Department of Biology and Geology, University of Almería, Almería 04120, Spain; “Pabellón de Historia Natural-Centro de Investigación de Colecciones Científicas de la Universidad de Almería” (PHN-CECOUAL), University of Almería, Almería 04120, Spain

**Keywords:** NUPTs, plastid DNA, genome evolution, plants, transposable elements, RNA genes

## Abstract

NUPTs are DNA sequences of plastid origin that are present in plant nuclear genomes in varying, though typically low, amounts. It is assumed that they are continuously formed and, due to their potentially mutagenic effect, they are removed at a constant turnover rate, which should result in an exponential decay of their age distributions and a negative correlation between age and size. However, these assumptions are based on analyses from a limited number of species and have never been explicitly tested. To gain insight into the mechanisms driving the origin and evolution of NUPTs, here we surveyed the plastid and nuclear genomes of 30 species representing the main angiosperm (flowering plants) lineages. By modeling the distribution of ages and sizes, examining their linear arrangement across the plastid genome, and statistically assessing spatial biases with respect to other genomic features, we showed that NUPTs are: (i) formed by both continuous and episodic mechanisms; (ii) unevenly represented across the plastid genome; (iii) consistently associated with certain classes of RNA genes, in particular rRNA, tRNA, and regulatory RNA genes; (iv) differentially contributing to structural genes; and (v) closer than expected to different superfamilies of transposons in a species-specific manner. Our results reveal the unexpected complexity in the mechanisms driving the origin of NUPTs, which not only involve their continuous formation but also episodic events, highlight their role as a major source of noncoding RNA genes and other genomic features, and provide a more complete picture of the different drivers of evolutionary change at the genome level.

Significance The putative role of DNA sequences of plastid origin, or NUPTs, in shaping the evolution of genome architecture and function remains less studied compared to that of transposable elements or gene and genome duplications. Here, we performed a comparative analysis of the plastid and nuclear genomes of 30 species representing the main angiosperm lineages in an attempt to elucidate the patterns underlying the mode of origin of NUPTs and assess the mechanisms driving their fate throughout evolutionary time. We revealed a hitherto unexpected complexity and diversity in the mechanisms underlying the origin and evolution of NUPTs, leading either to their rapid mutational decay or, alternatively, to their proliferation and subsequent fixation in the nuclear genome. Our results provide a more complete picture of the drivers of genome evolutionary change and emphasize the importance of accounting for the small but significant fraction of plastid DNA present in plant nuclear genomes.

## Introduction

Plant nuclear genomes are subject to a variety of mutational mechanisms that result in a wide range of sequence changes and structural rearrangements. After passing through the filter of selection and other evolutionary forces, these changes may be at the origin of evolutionary innovations and biological adaptations, largely determining phenotypic differences among organisms, populations, and species. Intensive research has therefore been devoted to understanding these mechanisms, two of which are thought to be the most important in generating genomic variation in plants. First, the proliferation of transposable elements (TEs) across the genome, which is responsible for most of the variations found in genome sizes and results in a wide variety of changes in plant gene expression and function (Lisch 2013; Schrader and Schmitz 2019; Tossolini et al. 2025). Second, gene and whole genome duplication (WGD), which provides an enormous source of raw genetic material that can be at the origin of novel or specialized gene functions and has been hypothesized to facilitate species diversification and domestication, as well as to confer an adaptive advantage during periods of environmental turmoil (Flagel and Wendel 2009; Carretero-Paulet and Fares 2012; Panchy et al. 2016; Carretero-Paulet and Van de Peer 2020). In contrast, the role of other potential drivers of genomic evolutionary change, such as DNA sequences of plastid origin, or NUPTs (Timmis et al. 2004), in shaping genome architecture and function has received less attention. NUPTs originate from the insertion of copies of DNA stretches of varying size derived from the plastid genome into the nuclear genome. Originally, they were thought to be associated with the transfer of massive amounts of DNA and genes from the protoorganelle’s bacterial genome to the nucleus during the endosymbiotic event that occurred ca. 1,600 million years ago, MYA (Timmis et al. 2004; Betts et al. 2018), and gave rise to the plastids. However, DNA segments of plastid origin are still being copied and relocated to the nuclear genome in a process that does not result in concomitant shrinking of the plastid genome. Therefore, NUPTs also provide a source of raw genetic material whose potential role in the origin of evolutionary innovations and biological adaptations at the genome level has yet to be properly addressed.

The sequence of events involved in NUPT formation has not been fully elucidated, but its insertion in the nuclear genome involves DNA breakage repair and is thus potentially mutagenic (Kleine et al. 2009). Most NUPTs are therefore expected to evolve neutrally or to be eventually deleterious and selected against, undergoing rapid decay over evolutionary time. This process probably entails various mechanisms, such as accumulation of point mutations, TE activity, the insertion of nonplastid DNA sequences, and replication slippage (Kleine et al. 2009; Michalovova et al. 2013; Zhang et al. 2020). Accordingly, the plastid DNA detected in most nuclear genomes typically represents only a small fraction of less than 0.1%, with very few showing more than 1% (Zhang et al. 2020). However, recent studies have reported up to 4% of nuclear DNA of plastid origin in *Moringa oleifera* (Ojeda-López et al. 2020; Marczuk-Rojas, Álamo-Sierra et al. 2024), raising questions about the molecular and evolutionary forces that may have acted on specific plant lineages to allow the fixation of substantial amounts of organellar DNA without compromising nuclear genome stability. Also, it follows that a rapid decay of NUPTs should result in a constant turnover rate, an assumption hitherto primarily supported by studies that reported an apparent exponentially decaying distribution of NUPTs' ages and a negative correlation between NUPTs' ages and sizes, which had not been statistically tested explicitly (Richly and Leister 2004; Michalovova et al. 2013; Yoshida et al. 2014). Indeed, a bimodal distribution of age estimates corresponding to episodic events of NUPTs' formation, together with a positive correlation between NUPTs' age and size, was recently reported in *M. oleifera* (Marczuk-Rojas, Álamo-Sierra et al. 2024).

It has been argued that NUPT formation could be conceived of as an opportunity for the origin and evolution of plastid-derived nuclear structural genes or gene regions (Sheppard and Timmis 2009). Nevertheless, (i) plastid-derived genes are believed to be dead upon arrival to the nucleus because they lack the regulatory regions needed for their proper transcription, with activation of genes of plastid origin in the nucleus considered a rare event (Kleine et al. 2009); (ii) only a few instances of incorporation of plastid DNA into pre-existing nuclear genes, either in exonic regions, i.e. exonization or in intronic ones, have been reported (Noutsos et al. 2007; Akbarova et al. 2010; Marczuk-Rojas, Salmerón et al. 2024); (iii) epigenetic regulation, and prominently DNA methylation and histone tail modification, has been associated with the transcriptional repression of integrated organellar DNA (Yoshida et al. 2019; Zhang et al. 2024); and (iv) albeit a role for NUPTs in the dissemination of regulatory elements in the promoter or enhancer of specific genes has been reported, resulting in a more efficient transcription (Ott and Chua 1990; Blanchard and Schmidt 1995; Mohan et al. 2016), overall transcription levels of structural genes affected by NUPTs in *M. oleifera* were in general not found to be different from those of their non-NUPTs counterparts (Marczuk-Rojas, Salmerón et al. 2024). In this species, NUPTs were also found to be strongly enriched among several classes of RNA genes.

To date, most studies of NUPTs have focused on a limited number of species; therefore, it has not been possible to conduct statistically supported analyses for assessing different assumptions regarding their origin and evolution and, ultimately, their impact on the evolution of nuclear genome architecture and function. Understanding the origin and evolution of NUPTs would certainly be improved by characterizing pools of bona fide NUPTs obtained from a large enough set of species. In an attempt to elucidate the patterns underlying the mode of origin of NUPTs and assess the mechanisms driving their fate throughout evolutionary time, we surveyed in the present study the nuclear and plastid genomes of 30 species representing the main angiosperm lineages. We focus on mathematically modeling their distribution of ages and sizes, examining their linear arrangement across plastid genomes, and statistically assessing spatial biases with respect to other genomic features. Our results reflect the existence of both episodic and ongoing mechanisms driving the origin and subsequent evolutionary and functional fate of NUPTs, which may act in a lineage-specific or shared manner, highlight the role of NUPTs in contributing to nuclear genome complexity, and provide a more complete picture of the different drivers of genome evolutionary change.

## Results

### Wide Variations in Plastid DNA Content Across 30 Angiosperm Nuclear Genomes

**Fig. 1.**
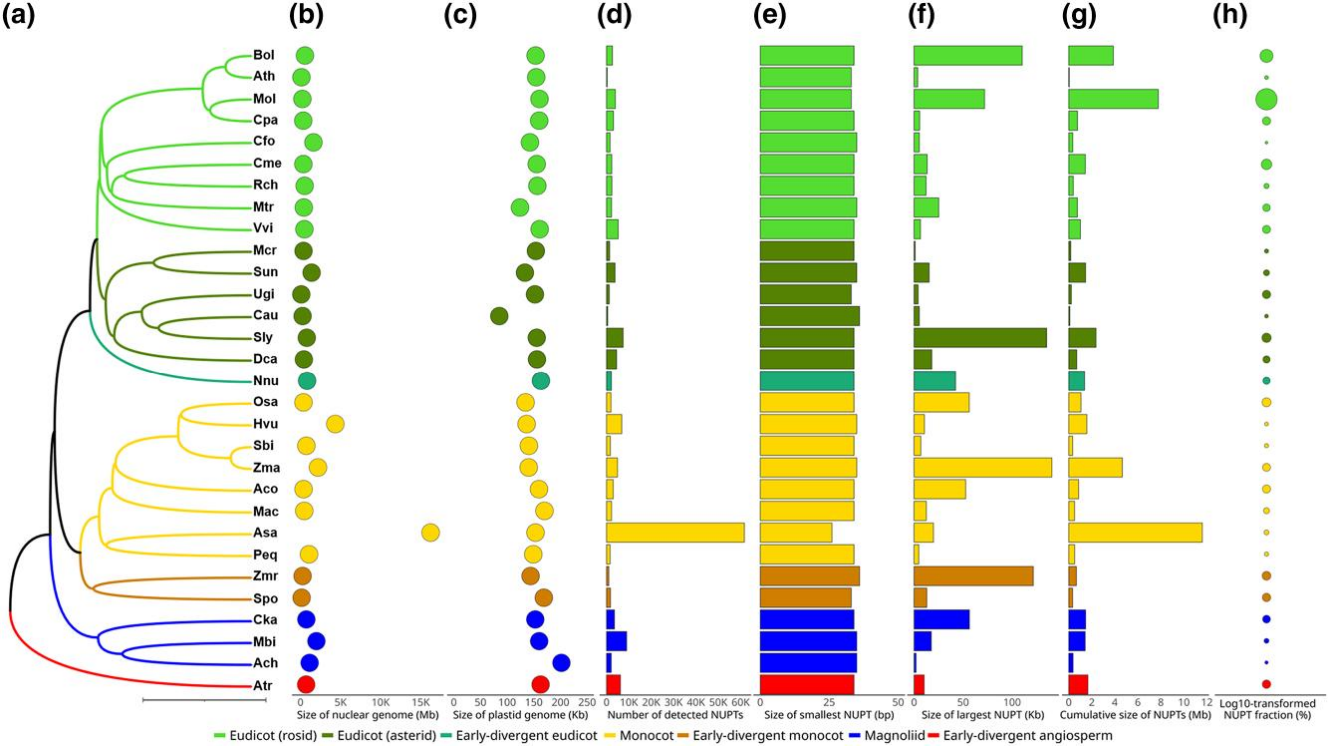
Exploratory analysis of 30 angiosperm nuclear and plastid genomes, as well as NUPTs, in a phylogenetic context. a) Phylogenetic tree depicting the evolutionary relationships among our 30-species set. Branches in the tree are proportional to evolutionary time, with the scale bar representing 100 MYA. b) Size of nuclear genome assembly. c) Size of plastid genome assembly. d) Total number of NUPTs. e) Minimum size of NUPTs. f) Maximum size of NUPTs. g) Cumulative size of NUPTs. h) Fraction of NUPTs in the nuclear genome. The values were log10-transformed for better visualization. The names of the species are abbreviated as in Table 1.

**Fig. 2.**
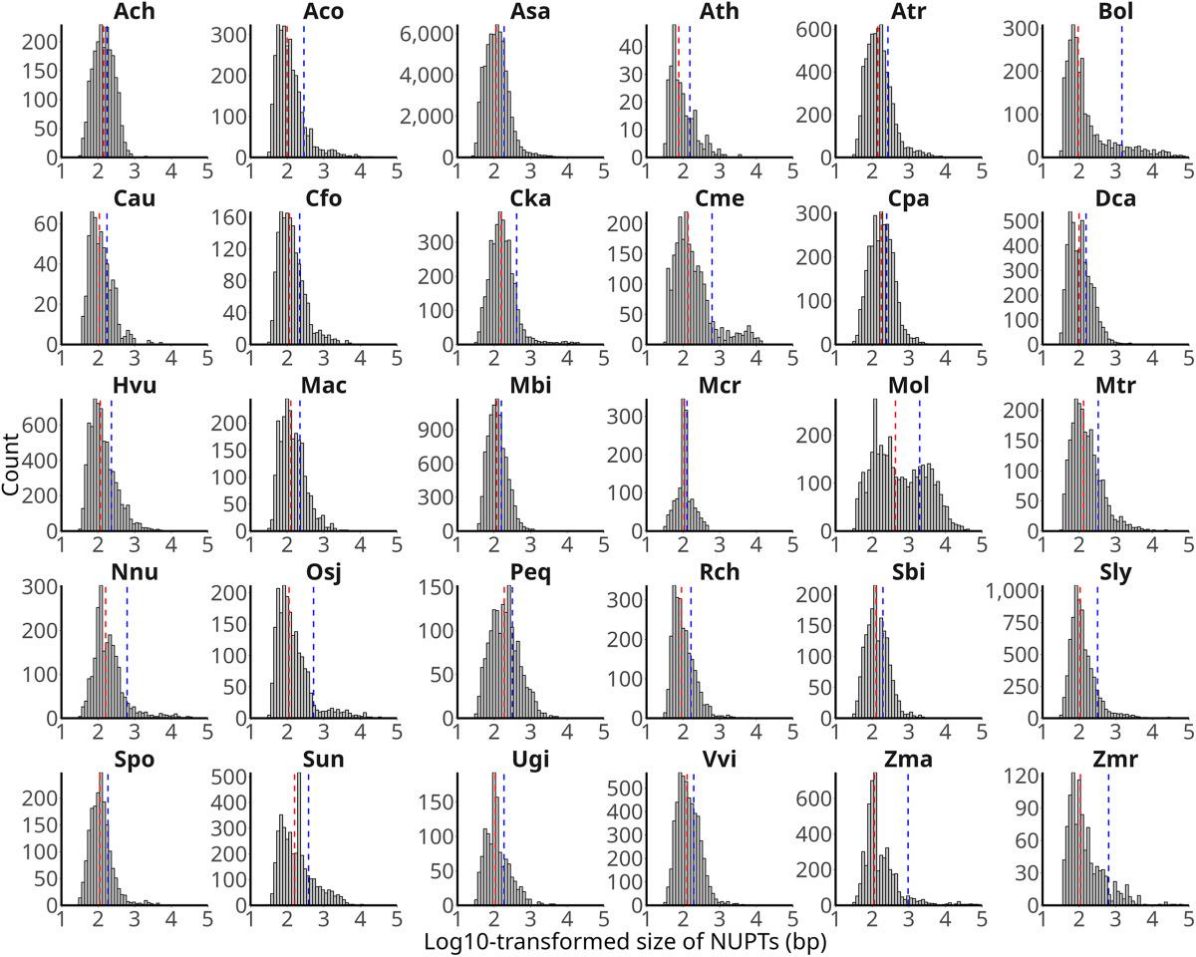
Histograms of log10-transformed NUPT sizes. The mean and median are indicated as blue and red dashed lines, respectively. The names of the species are abbreviated as in Table 1.

After filtering out redundant hits resulting from Inverted Repeat (IR) regions of the plastid genome and spurious hits corresponding to low-complexity regions, a total of 152,513 bona fide NUPTs were detected in 30 species representing the major angiosperm lineages (Table 1 and Fig. 1a). The species were also selected based on genome assembly levels (contig, scaffold, and chromosome) and representativeness of long- or short-read genome sequencing technologies, as well as differences in nuclear and plastid genome sizes (showing 160- and 2.38-fold variations from 100 to 16,243 Mb and 85 to 202 kb, respectively) and the reported fraction of repeat DNA (ranging from 16.23% to 91.3%) (Table 1, Fig. 1a to c and Table S1). The number of NUPTs identified per species varied widely from less than 1,000 in *Arabidopsis thaliana* and *Cuscuta australis* to more than 60,000 in *Allium sativum* (Fig. 1d and Table S2). For most species, the distribution of NUPT sizes was right-skewed (Fig. 2 and Table S3), with the smallest NUPTs encompassing a narrow range between 26 and 36 bp (Fig. 1e and Table S3) and the largest NUPTs oscillating between 1.1 and 141 kb (Fig. 1f and Table S3). An individual NUPT of 141 kb in length, spanning the entire plastid genome (Maier et al. 1995) and the largest to date, was detected in maize. This NUPT seems to be of recent origin, as suggested by the high percent of sequence identity shown (99.61%). The cumulative size of NUPTs was featured by an extensive range of values that encompassed four orders of magnitude and exceeded in all species the size of their respective donor plastid genomes, except *A. thaliana* (Fig. 1g and Tables S2). The total fraction of the genome occupied by NUPTs ranged between 0.02% and 0.70% in all species but *M. oleifera*, with 3.29% (Fig. 1h and Table S2).

**Table 1:** Angiosperm species selected for this study

Species	Abbreviation	Lineage	Species	Abbreviation	Lineage
*Allium sativum*	Asa	Monocot	*Mesembryanthemum crystallinum*	Mcr	Dicot (asterid)
*Amborella trichopoda*	Atr	Early-divergent angiosperm	*Moringa oleifera*	Mol	Dicot (rosid)
*Ananas comosus*	Aco	Monocot	*Musa acuminata ssp. Malaccensis*	Mac	Monocot
*Annona cherimola*	Ach	Magnoliid	*Nelumbo nucifera*	Nnu	Early-divergent dicot
*Arabidopsis thaliana*	Ath	Dicot (rosid)	*Oryza sativa ssp. Japonica*	Osj	Monocot
*Brassica oleracea HDEM*	Bol	Dicot (rosid)	*Phalaenopsis equestris*	Peq	Monocot
*Carica papaya*	Cpa	Dicot (rosid)	*Rosa chinensis cv. Old Blush*	Rch	Dicot (rosid)
*Cephalotus follicularis*	Cfo	Dicot (rosid)	*Selenicereus undatus cv. Guanhuabai*	Sun	Dicot (asterid)
*Cinnamomum kanehirae*	Cka	Magnoliid	*Solanum lycopersicum*	Sly	Dicot (asterid)
*Cucumis melo cv. DHL92*	Cme	Dicot (rosid)	*Sorghum bicolor*	Sbi	Monocot
*Cuscuta australis*	Cau	Dicot (asterid)	*Spirodela polyrhiza*	Spo	Early-divergent monocot
*Daucus carota*	Dca	Dicot (asterid)	*Utricularia gibba*	Ugi	Dicot (asterid)
*Hordeum vulgare cv. Morex*	Hvu	Monocot	*Vitis vinifera*	Vvi	Dicot (rosid)
*Magnolia biondii*	Mbi	Magnoliid	*Zea mays cv. B73*	Zma	Monocot
*Medicago truncatula*	Mtr	Dicot (rosid)	*Zostera marina*	Zmr	Early-divergent monocot

The assembly version and source, together with other information, can be found in Table S1 for both nuclear and plastid genomes.

### Modeling the Ages of NUPTs Supports both Continuous and Episodic Modes of Origin

It has been commonly assumed that NUPTs are being formed and eliminated continuously at a constant rate, which should result in an exponential distribution of age estimates (Yoshida et al. 2014; Chen et al. 2015; Zhang et al. 2020). However, this assumption had not been explicitly tested, and recently, bimodal and unimodal distributions of age estimates corresponding to episodic events of NUPTs' formation were reported in *M. oleifera* and *Ziziphus jujuba*, respectively (Marczuk-Rojas, Álamo-Sierra et al. 2024; Yang et al. 2024). Age distribution-based methods are routinely used to model the evolution of gene duplicates and identify peaks corresponding to episodic events of WGD, where all genes duplicate at the same evolutionary timepoint, over the background exponential distribution of ages corresponding to ongoing gene duplication and loss events affecting small genomic regions (Maere et al. 2005; Vanneste et al. 2013). The number of synonymous substitutions per synonymous site, *K*_s_, between the coding sequences of gene duplicates has been the most used estimate of age since duplication, because synonymous substitutions do not result in amino acid changes and are therefore assumed to accumulate neutrally or nearly neutrally at an approximate constant rate throughout evolutionary time (Kimura 1977). Nevertheless, several concerns have been raised about the use of nucleotide substitution rates as a proxy for the age of gene duplicates. These concerns result mainly from substitutional saturation effects and the stochastic nature of nucleotide substitutions (Vanneste et al. 2013), which may in turn result in spurious peaks in older duplicates (*K*_s_ > 1 to 2) incorrectly interpreted as ancient WGD events. Taking this into account, and under the assumption that nucleotide substitutions in NUPTs accumulate neutrally at a rate proportional to the evolutionary time elapsed since their origin, the corrected number of substitutions per nucleotide site, or *K*, was used here as an estimate of NUPT relative age (Fig. S1 and Table S4). Our *K* estimates can be considered as a reliable proxy for the age of NUPTs, as (i) they were corrected to account for multiple substitutions at the same site, and (ii) they were below 0.38 in all cases. In addition, univariate mixture modeling techniques have been proven successful in identifying peaks in the *K*_s_ distribution of gene duplicates corresponding to WGD events (Maere et al. 2005; Tiley et al. 2018). However, mixture normal models often overfit the number of peaks, leading to overestimates of the number of ancient WGDs.

**Fig. 3.**
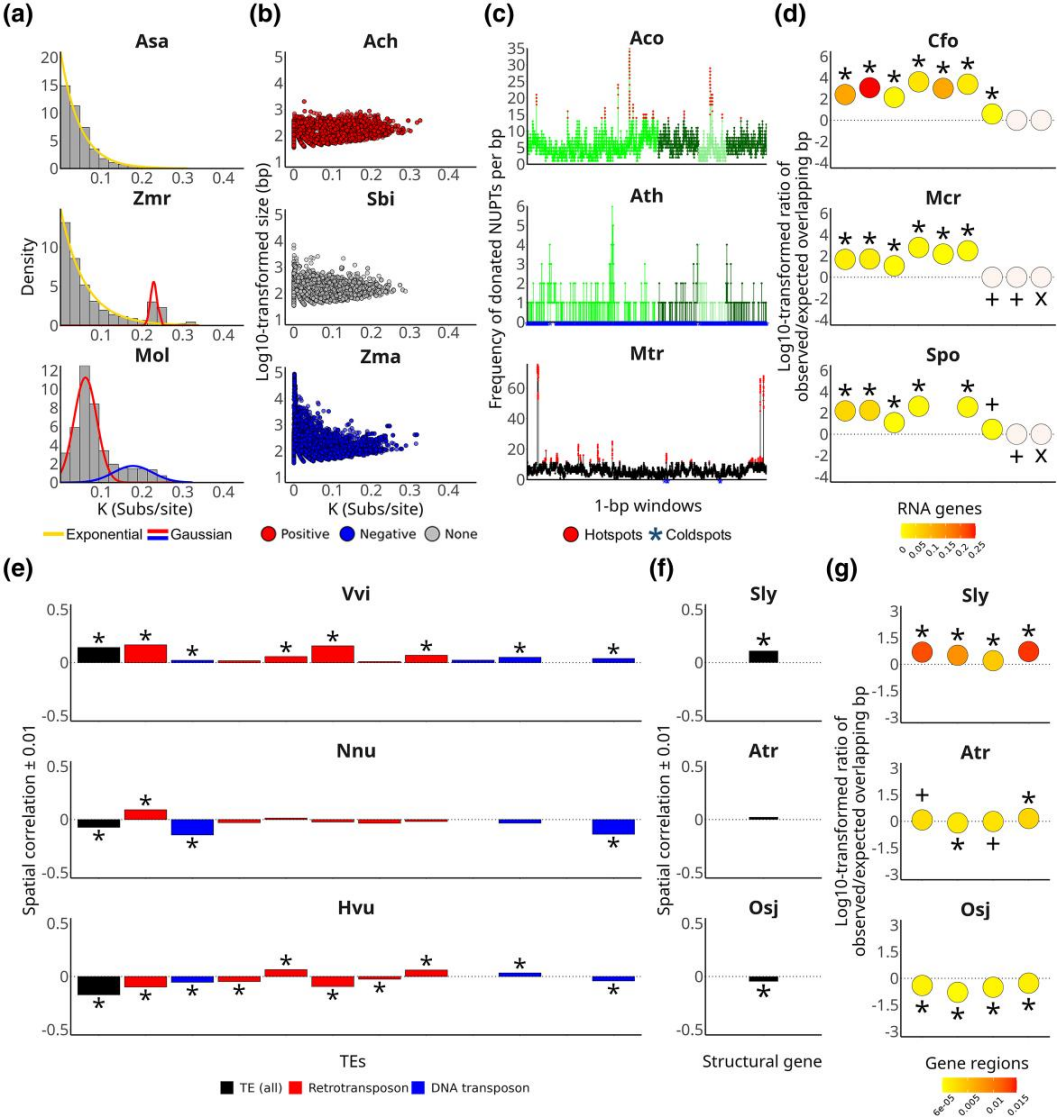
Spatial and temporal analysis of NUPTs in angiosperm species. Only results for representative species are shown; for the complete graphical representations of the analyses, see Figs. S2 to S5, S7, S9, and S10. a) Modeling the distributions of NUPTs' ages (using *K* as a proxy). Density curves representing the best-fitting distribution model, colored according to the components of the selected model, are shown overlaying the corresponding histograms. b) Correlation analysis between NUPTs' ages and sizes. c) Distribution of NUPTs across the plastid genome. The frequencies of NUPTs per bp are displayed as line-dot plots colored according to the four regions typically featuring the plastid genome, which are represented in the *x* axis, except for Mtr, which did not show the canonical quadripartite structure. From left to right: Large Single Copy, Inverted Repeat A, Small Single Copy, and Inverted Repeat B. Hotspots and coldspots across the plastid genome. d) Overlap analysis between NUPTs and RNA genes. RNA genes are depicted as circles whose positions on the *y* axis and colors represent two different overlap tests, i.e. counts of overlapping bp and Jaccard index, respectively. The significance of the tests is indicated as follows: (*) significant according to both, (+) only significant according to the test based on the counts of overlapping bp, and (x) only significant according to the test based on the Jaccard index. From left to right: eukaryotic rRNA, prokaryotic rRNA, nuclear tRNA, plastid tRNA, mitochondrial tRNA, self-splicing intron RNA (groups I and/or II), regulatory RNA, spliceosomal RNA, and other RNA. e) Distance analysis based on RDT tests between NUPTs and TEs. TEs, as a whole and classified by classes and orders, are displayed as colored bars whose heights correspond to a measure of the spatial correlation between NUPTs and them, i.e. their tendency to be found closer (positive correlation) or farther than expected (negative correlation). Significant correlations are indicated with an asterisk. From left to right: TEs as a whole, TE class I/Retrotransposon, TE class II/DNA transposon, Retrotransposon/DIRS, Retrotransposon/LINE, Retrotransposon/LTR, Retrotransposon/PLE, Retrotransposon/SINE, DNA transposon/Crypton, DNA transposon/Helitron, DNA transposon/Maverick, and DNA transposon/TIR. f) Distance analysis based on RDT tests between NUPTs and structural genes. g) Overlap analysis between NUPTs and structural genes. From left to right: promoter, exon, intron, and terminator. The names of the species are abbreviated as in Table 1.

To shed light on the mode of origin of NUPTs in our 30-species set, four different univariate mixture models were fitted to their respective distributions of age estimates based on *K* values. First, the continuous gain and loss of NUPTs was modelled using an exponential distribution. Second, to detect peaks corresponding to putative episodic formation events of NUPTs, while reducing the risk of overfitting, the number of peaks, single Gaussian models, and two-component Gaussian mixture models were fitted. Finally, a mixture model combining an exponential distribution with a single Gaussian was used to model background NUPT gain and loss while accounting for episodic origin. For each nuclear genome, 15 replicates were generated, and the best-fitting model in each replicate was evaluated using the Bayesian Information Criterion (BIC). A mixture of exponential and Gaussian distributions of NUPT relative ages was identified as the most frequent best-fitting model in 26 species (Fig. 3a and Fig. S2). According to the posterior probabilities of assigning a NUPT to either one or another component, the episodic mode of formation contributed to the global pool of NUPTs in all of them, but *A. sativum*, for which the exponential only was selected as the best-fitting model (Table S5). In the remaining four species, namely, *Amborella trichopoda*, *Magnolia biondii*, *M. oleifera*, and *Utricularia gibba*, a Gaussian mixture with two components was found as the best-fitting model (Fig. 3a and Fig. S2).

### Correlation Analysis Reveals Mutational Decay Is Not the Only Evolutionary Fate of NUPTs

Another common assumption about the evolution of NUPTs is that they are subject to rapid decay and fragmentation, and eventually loss, due to the continuous accumulation of nucleotide substitutions and indels (Richly and Leister 2004; Michalovova et al. 2013; Yoshida et al. 2014). This assumption would result in a negative correlation between NUPTs' age and size, i.e. the older the NUPT, the shorter it should be. To verify this assumption, we examined the correlation between NUPTs' distributions of age and size in our 30-species set. To account for non-normality in both variables, we implemented Kendall's rank-based correlation test (Fig. 3b and Fig. S3). Only six species exhibited significant negative correlations between NUPTs' age and size, while 19 species showed significant positive correlations; the remaining five species did not show a significant correlation at the 0.05 significance level (Fig. 3b). The positive correlation was evenly distributed among genome assemblies regardless of whether they were obtained using short (nine) or long-read sequencing technologies (ten), the latter including three obtained using a combination of short and long-read sequencing technologies. The analysis was replicated using Spearman's rank-based correlation tests, yielding similar results in all species (Fig. S3).

### Heterogeneous Distribution of NUPTs Across the Plastid Genome

Previous studies have reported that each region of the plastid genome was represented in NUPTs with a similar frequency, i.e. the distribution of NUPTs' sites of origin was homogeneous throughout the plastid genome (Matsuo et al. 2005; Yoshida et al. 2014). However, recent findings have revealed exceptions to this trend in *Asparagus officinalis* and *M. oleifera* (Li et al. 2019; Marczuk-Rojas, Álamo-Sierra et al. 2024). These exceptions manifest as hotspots of NUPTs, i.e. specific plastid genome regions significantly overrepresented among NUPTs, defined here as outliers of the Interquartile Range (IQR), and coldspots, defined as plastid regions not represented in any NUPT. Hotspots of NUPTs were detected in the plastid genomes of 29 species, of which 19 also contained coldspots; the only species containing coldspots and apparently lacking hotspots was *A. thaliana* (Fig. 3c, Fig. S4, and Table S6). The fraction of the plastid genome occupied by hotspots varied largely between 0.13% in *M. oleifera* and 16.40% in *Cucumis melo*, whereas the fraction corresponding to coldspots spanned between 0.001% in *Hordeum vulgare* and 78.14% in *A. thaliana* (Table S6). Hotspots and coldspots could be detected in any of the four regions typically featuring the plastid genome (Large Single Copy or LSC, the IRs, and Small Single Copy or SSC) (Ruhlman and Jansen 2021) in all species except *Medicago truncatula*, whose plastid genome did not show the canonical quadripartite structure (Choi et al. 2022). Next, we studied whether the fraction of the plastid genome occupied by coldspots or hotspots of NUPTs correlated with the fraction of plastid DNA present in the nuclear genome by using two rank-based correlation tests, Kendall's and Spearman's, respectively. A negative correlation between the fraction of coldspots and the fraction of plastid DNA in the nuclear genome was found, which resulted significant under both tests (Kendall's: τ = −0.38 and *P* = 4.39 × 10^−3^; Spearman's: ρ = −0.52 and *P* = 3.41 × 10^−3^). In contrast, no significant correlation was found between the fraction of hotspots in the plastid genome and the fraction of plastid DNA in the nuclear genome (Kendall's: τ = −0.02 and *P* = 0.9; Spearman's: ρ = −0.02 and *P* = 0.92).

### NUPTs Are Strongly and Consistently Enriched Among Different Classes of RNA Genes

A spatial analysis performed in *M. oleifera* revealed that NUPTs were consistently overrepresented among different classes of RNA genes (Marczuk-Rojas, Salmerón et al. 2024). Here, we sought to identify analogous enrichments in the distribution of NUPTs across the nuclear genomes of our 30-species set. To employ a uniform definition of RNA genes, the nonstructural gene space of all 30 nuclear genomes was first reannotated. We then characterized the contribution of NUPTs to every class of RNA genes by examining their overlap using two different approaches.

First, comparing observed versus expected counts of overlapping bp between NUPTs and every other genomic feature. As described in *M. oleifera*, the observed counts of overlapping bp were consistently higher than expected in all the remaining 29 species for eukaryotic and prokaryotic rRNA genes, nuclear, mitochondrial, and plastid tRNA genes, and self-splicing RNA introns from groups I and/or II (Fig. 3d, Fig. S5, and Table S7). The opposite trend was observed for spliceosomal RNA genes and other RNA genes, which had zero total overlap, i.e. zero overlapping bp, in all species but *Cinamomum kanehirae* for the former and *A. sativum* for both; these trends were statistically significant in most cases (Fig. 3d, Fig. S5, and Table S7). The only class of RNA genes that did not show a common trend among species was that of regulatory RNA genes taken as a whole, for which the observed counts of overlapping bp were significantly higher than expected in 25 of 30 species, whereas the remaining five displayed the opposite trend (Fig. 3d, Fig. S5, and Table S7). We next repeated the overlap analysis between NUPTs and regulatory RNA genes at the subclass level. NUPTs were consistently and specifically overrepresented among *iron stress-repressed RNA* (*isrR*) genes in all 25 species where they were detected, while they were found not to overlap or be underrepresented among the remaining four subclasses of regulatory RNA genes in all species but *A. thaliana* (Fig. S6 and Table S8); again, these trends were statistically significant in most cases. Second, we implemented a different approach to detect spatial overlap between NUPTs and RNA genes based on the Jaccard test, and the results obtained were mostly consistent with those resulting from overlapping bp counts (Fig. 3d, Figs. S5 and S6, and Tables S7 and S8).

### NUPTs Colocalize with Different Superfamilies of TEs in a Species-Specific Manner

Previous studies supported that NUPTs are found closer to specific superfamilies of TEs than expected (Richly and Leister 2004; Michalovova et al. 2013; Wang et al. 2022; Marczuk-Rojas, Salmerón et al. 2024). To confirm this, we implemented the relative distance test (RDT) between NUPTs and TEs, which were de novo reannotated in our set of 30 nuclear genomes. When taken as a whole, TEs did not show a common spatial correlation trend with NUPTs, i.e. 16 and 14 species showed distances between NUPTs and TEs closer to and farther than expected, respectively, with tests being statistically significant in all cases except *Spirodela polyrhiza* (Fig. 3e, Fig. S7, and Table S9).

The analysis was repeated by separately considering the two major classes of TEs, i.e. class I (retrotransposons) and class II (DNA transposons). A significant positive correlation was found between NUPTs and retrotransposons in 16 species and with DNA transposons in three, whereas 10 and 22 species featured a significant negative correlation with the former and the latter, respectively (Fig. 3e, Fig. S7, and Table S9). We next examined the spatial correlation of NUPTs with TEs at the order and superfamily levels, respectively. Again, we did not observe a common trend toward NUPTs among the different TE orders (Fig. 3e, Fig. S7, and Table S9). Likewise, among TE superfamilies, none was found to follow a consistent pattern of positive or negative spatial correlation with NUPTs, with every species exhibiting significant positive correlations between them and at least one TE superfamily, with an apparent preference toward retrotransposon ones (Fig. S8 and Table S9). Significant positive spatial correlation between NUPTs and at least one retrotransposon superfamily was found in all species but *Mesembryanthemum crystallinum* and *Rosa chinensis*, whereas up to six species did not show any evidence of positive spatial correlation with any superfamily of DNA transposons. In total, 120 superfamilies that included 14 of the 16 identified superfamilies of retrotransposons and 56 superfamilies that encompassed 9 of the 12 identified DNA transposons exhibited a significant positive spatial correlation with our 30-genome NUPTs' dataset, respectively.

### The Spatial Arrangement of NUPTs Relative to Structural Genes also Exhibits Species-Specific Biases

Specific genomic features are known to influence the function of structural genes in a distance-based manner. For example, transcription factor binding sites, TFBSs, are found in the promoter and enhancer regions of structural genes, commonly in proximity to their coding regions; therefore, the distances between TFBSs and structural genes tend to be smaller than expected by chance (Yu et al. 2016). We check for putative similar biases in the spatial arrangement of NUPTs with respect to structural genes by implementing the RDT test. NUPTs and structural genes were found to be significantly closer than expected in 19 species, whereas only four showed the opposite trend (Fig. 3f, Fig. S9, and Table S10).

The above results may indicate the involvement of NUPTs in the acquisition of gene regulatory sequences, as has been previously shown (Ott and Chua 1990; Blanchard and Schmidt 1995; Mohan et al. 2016). To test whether this was the case, we first performed an overlap analysis based on counts of overlapping bp between NUPTs and promoter and terminator regions, defined here as the 1-kb sequences adjacent to the first and last exon, respectively. The results, shown in Fig. 3g, Fig. S10, and Table S10, indicate that in most species the observed counts of overlapping bp were significantly higher than expected for promoters (25) and terminators (27), including all 19 species that were also featured by having NUPTs significantly closer to structural genes according to the RDT. Almost identical results were obtained when we implemented the overlap analysis based on the Jaccard index (Fig. 3g, Fig. S10, and Table S10).

Moreover, although NUPTs inserted into coding regions of nuclear structural genes are expected to be deleterious because their insertion could disrupt their open reading frames, in a few cases, they have been reported to reshape structural genes by adding extra coding regions, by integrating into pre-existing exons, or, more frequently, by contributing new single-exon structural genes entirely of plastid origin (Noutsos et al. 2007; Akbarova et al. 2010; Chen et al. 2015; Pinard et al. 2019; Marczuk-Rojas, Salmerón et al. 2024). Indeed, the overlap analysis between NUPTs and exons yielded contrasting results among the 30 species, with 16 of them showing an observed number of overlapping bp significantly higher than expected, while 13 featured the opposite trend (Fig. 3g, Fig. S10, and Table S10). The overlap analysis was repeated using the Jaccard test, mostly corroborating our previous observations, except for four species that did not show significant results (Fig. 3g, Fig. S10, and Table S10). Similarly, disruptions affecting intronic regions can also be deleterious since they may alter pre-mRNA alternative splicing or gene expression activation (Morello and Breviario 2008; Gallegos and Rose 2015). However, NUPTs have been detected more frequently within introns than exons (Blanchard and Schmidt 1995; Noutsos et al. 2007; Akbarova et al. 2010; Marczuk-Rojas, Salmerón et al. 2024). In 19 of our 30-species set, NUPTs were significantly associated with introns, with tests based on overlapping bp counts and the Jaccard index yielding similar results (Fig. 3g, Fig. S10, and Table S10).

## Discussion

We attempted here to elucidate the tempo and mode of origin of NUPTs, as well as the evolutionary and functional fate of plastid DNA in their new genomic environment by surveying a genomic dataset of 30 angiosperm species. Many NUPTs are expected to assimilate rapidly into their new genomic environment through the accumulation of mutations that result in the amelioration of the plastid DNA sequence to the nucleotide composition of its host chromosome. Consistent with this, *K* estimates for NUPTs were in all cases below 0.38, corresponding to a minimum sequence identity with the plastid genome of 69% and likely indicating their relatively recent origin. In turn, it has been reported that long, very recent integrants of organellar DNA showing high sequence identity may be absent from nuclear genome assemblies (Fields et al. 2022), especially those based on short-read sequencing technologies. Consequently, the fraction of plastid DNA present in the nuclear genomes estimated on the basis of standard alignment methods may miss very old and very young NUPTs and can therefore be considered an underestimate. Notwithstanding this, we were still able to detect a large enough pool of NUPTs in each of the selected species to conduct a rigorous and statistically supported analysis. We discarded NUPTs corresponding to spurious alignments involving low-complexity regions present in the plastid genomes, as well as redundant NUPTs resulting from IR regions, and obtained sets of NUPTs free of false positives likely representing true homology. This filtering step also allowed on to unambiguously identify coldspots and hotspots of NUPTs in plastid genomes. The diverse, yet occasionally large, fraction of the plastid genome corresponding to hotspots, and its lack of correlation with the total fraction of plastid DNA present in the nuclear genome, might suggest that they cannot be considered hyperdonating regions featured by their preferential copy and translocation to the nucleus, and that most NUPTs actually originate from postinsertion duplications of specific NUPTs. Conversely, a slight, though significant, negative correlation was detected between the fraction of coldspots and NUPTs, suggesting they could correspond to plastid regions either less prone to their copy and relocation to the nuclear genome or deleterious, and therefore selected against. Very short coldspots, especially those formed by a few bp, might also be involved in the formation of NUPTs not detected by current alignment-based methods.

Due to their mutagenic and thus potentially deleterious nature, it has traditionally been assumed that NUPTs undergo rapid decay after insertion as a result of various mutational mechanisms, being eliminated rapidly from the nuclear genome and eventually reaching a state of equilibrium between formation and removal (Matsuo et al. 2005; Sheppard and Timmis 2009). Such continuous formation and subsequent rapid elimination of NUPTs under a constant turnover rate should result in (i) an exponentially decaying curve of NUPTs’ age distributions; and (ii) a negative correlation of NUPTs' age and size. Nonetheless, in our study, only one species was found to show the best fit with the exponential-only distribution, while most species showed a better fit with a model defined by a mixture of an exponential and a Gaussian component, the latter being suggestive of episodic formation. Furthermore, our correlation analysis revealed that many species exhibited a positive correlation between NUPTs' age and size, consistent with the occurrence of postinsertion duplication events or the long-term retention of large NUPTs. One could argue that the expected underrepresentation of long recent NUPTs among genome assemblies obtained using short-read sequencing technologies results in the observed correlation patterns (Fields et al. 2022). However, in our analysis, positive correlation was evenly distributed among genome assemblies regardless of the sequencing technology.

This episodic mode of NUPT formation would lead to their proliferation and eventual retention over longer evolutionary times, i.e. their fixation in the nuclear genome. This raises the question of the potential adaptive role of NUPTs in genome evolution. Certain environmental stresses are known to increase the frequency of NUPT formation (Cullis et al. 2009; Wang et al. 2012), and NUPTs have been attributed to have a potential role in enhancing environmental adaptation (Ma et al. 2020). Therefore, an interesting hypothesis to test is whether bursts of episodic NUPT formation events correlate with stressful and changing environments. Such a correlation has been shown for WGD, which confers an adaptive advantage during extinction events (Carretero-Paulet and Van de Peer 2020). This would require the simultaneous formation of NUPTs in all species around specific evolutionary time periods. However, the strong variation in the relative ages, counts, and sizes of NUPTs found among species suggests that they form in episodic bursts specific to lineages. In addition, although we did not find a strong, consistent pattern of colocalization between NUPTs and specific TE classes, orders, or superfamilies, all species showed preferential association with at least one TE superfamily, typically of retrotransposons. Retrotransposons are also known to be activated by multiple environmental stresses (Ito 2022) and transpose through a copy-and-paste mechanism, which can explain the dispersion and proliferation of NUPTs throughout the nuclear genome after their insertion. Thus, the differences in the transposition mechanism, activation state, and stress responsiveness of the specific TE superfamilies with which NUPTs preferentially colocalize likely play a prominent role in determining the ultimate fate of NUPTs, whether they are eliminated or proliferate, and ultimately explain species-specific variations in the plastid DNA content of the nuclear genome.

Long-term fixation of NUPTs shows spatial biases with respect to different genomic features, which may be species-specific or shared depending on the genomic feature considered. For example, in 19 of 30 species, NUPTs are significantly closer to structural genes than expected by chance, reflecting (i) the differential action of purifying selection in purging away potentially deleterious insertions of NUPTs into structural genes, or (ii) the opportunity of NUPTs for evolutionary innovation by providing new gene, gene regions, or regulatory elements. In contrast, and as previously shown in *M. oleifera* (Marczuk-Rojas, Salmerón et al. 2024), some classes of RNA genes appear consistent and significantly enriched for NUPTs in all species, including (i) rRNA and tRNA genes involved in protein biosynthesis; (ii) *isrR* genes, a subclass of iron-deficiency-responsive regulatory RNA genes formerly considered exclusive to cyanobacteria whose function in plants remains unknown to this date (Dühring et al. 2006); or (iii) self-splicing RNA introns, capable of performing both self-splicing and retrotransposition (Novikova and Belfort 2017). Albeit RNA genes derived from NUPTs are not expected to be functional because they lack promoters and other essential elements required for their proper expression, a significant fraction of them was found to be functionally expressed in *M. oleifera* (Marczuk-Rojas, Salmerón et al. 2024). In this respect, it is noteworthy that specific superfamilies of retrotransposons are known to provide promoter regions, enabling the expression of neighboring genes (Panchy et al. 2016; Dubin et al. 2018). Furthermore, sequences derived from the plastid genome could adapt to the new nuclear environment through concerted evolution; this process is known to occur among nuclear *rRNA* and *tRNA* genes, resulting in their remarkable conservation at the sequence level (Eickbush and Eickbush 2007). *tRNA* genes are also known to exhibit remarkable evolutionary plasticity (Yona et al. 2013). This plasticity may facilitate plastid-derived *tRNA* genes to rapidly evolve at the sequence level to adapt to novel translational demands in the nucleus. Altogether, it is tempting to speculate that NUPTs contribute to the nuclear pool of rRNA and tRNA genes and thus to the protein biosynthetic machinery. However, some rRNA and tRNA genes may also evolve extraribosomal functions, as discussed by Marczuk-Rojas, Salmerón et al. (2024).

In conclusion, the present study has revealed a hitherto unexpected complexity and diversity in the mechanisms underlying the origin and evolution of NUPTs, leading either to their rapid mutational decay or, alternatively, to their proliferation and subsequent fixation in the nuclear genome. Plastid DNA emerges as a major source of nonstructural genes, especially RNA genes involved in protein biosynthesis or playing regulatory functions, thus contributing decisively to the evolution of plant nuclear genome architecture and function. Our results provide a more complete picture of the drivers of genome evolutionary change and emphasize the importance of accounting for the small yet significant amount of plastid DNA present in plant nuclear genomes.

## Material and Methods

### Detection of NUPTs and Species Phylogenetic Tree

The nuclear genomes of 30 angiosperm species were scanned for NUPTs using the BLASTN local alignment tool from the BLAST+ program package v2.12.0+ (Camacho et al. 2009), with their corresponding plastid genomes as queries, and parameters as reported by Marczuk-Rojas, Álamo-Sierra et al. (2024). To mask low-complexity regions present in the plastid genome that might result in spurious alignments wrongly interpreted as NUPTs, BLASTN was run by turning on the -dust setting (−dust yes). Every single BLASTN hit was considered an individual NUPT. To correct for redundancy in NUPTs resulting from the duplicated IR regions typically present in plastid genomes, duplicated BLASTN hits involving IR regions were counted only once, by selecting one or another IR region in a random manner. IRs were identified in every plastid genome using IRplus (https://irscope.shinyapps.io/IRplus/) (Díez Menendez et al. 2023). Hotspots of NUPTs were detected by determining the frequency of NUPTs across the plastid genome in windows of 1 bp in length and identifying large outliers within that frequency through the IQR rule. The IQR is the difference between the third quartile (Q3) and the first quartile (Q1) of a dataset. To find large outliers, any data point must fall above Q3 + 1.5 * IQR. In turn, 1-bp plastid regions not represented in any NUPT were defined as coldspots. A species tree depicting evolutionary relationships among the selected species was generated with Itol v6 (Letunic and Bork 2024) using the topology and divergence times retrieved from the TimeTree v5 database (Kumar et al. 2022).

### Genome Structural Reannotation

To obtain a uniform annotation of the different genomic features beyond structural genes present in our set of 30 species, we implemented the following pipeline. First, repeat identification and classification in the nuclear genomes was performed according to (Chang et al. 2022). Briefly, RepeatModeler v2.0.4 (Flynn et al. 2020) was used for the identification step, and unidentified TEs were further examined through DeepTE (Yan et al. 2020 ). Subsequently, the output data from RepeatModeler and DeepTE were utilized as a custom repeat library for RepeatMasker v4.1.5 (http://www.repeatmasker.org) to detect and classify repeats in the nuclear genomes with the default parameters. Finally, TEs were reclassified following the classification system reported by Wicker et al. (2007) to unify the respective nomenclatures employed by RepeatModeler and DeepTE, resulting in 2 classes, 9 orders, and 28 superfamilies of TEs.

Second, RNA genes were detected by scanning the nuclear genomes with the RFAM v14.10 database of noncoding RNA families (Griffiths-Jones et al. 2003) using the command cmscan from Infernal v1.1.5 (Nawrocki and Eddy 2013 ). Furthermore, tRNA genes found by Infernal were completed by merging with the annotation obtained from running tRNAscan-SE v2.0.10 (Chan et al. 2021) with the option –E activated to search for tRNA from the *eukarya* domain. Every tRNA gene identified was further classified as nuclear, mitochondrial, or plastid according to the annotation of the best hit resulting from BLASTN v2.12.0+ (Camacho et al. 2009) searches of a database of *A. thaliana* tRNA genes retrieved from PltRNAdb (https://bioinformatics.um6p.ma/PltRNAdb/) (Mokhtar and Allali 2022), selecting a word size of 11 and an *E*-value of 10^−2^ as settings. Our genome reannotation did not incorporate long noncoding RNA genes because all available methods are based on RNA-seq data, and a uniform annotation across our species was deemed unfeasible.

### Univariate Modeling of NUPTs' Distributions of *K*

The number of substitutions per nucleotide site, *K*, of every NUPT sequence was calculated based on the corresponding pairwise BLASTN alignment. To account for multiple nucleotide substitutions per site, the resulting *K* values of NUPTs were corrected by the one-parameter Jukes–Cantor, 69 substitution model (JC69) (Jukes and Cantor 1969), which had been employed in previous studies (Matsuo et al. 2005; Namasivayam et al. 2023). R scripts retrieved from (https://github.com/gtiley/Ks_plots) (Tiley et al. 2018), originally designed to detect episodic WGD events from distributions of synonymous substitutions per synonymous site, *K*_s_, of gene duplicates, were employed here to fit four different univariate models, i.e. exponential, Gaussian, Gaussian mixture with two components, and exponential-Gaussian mixture, to the *K* distributions of NUPTs; 15 replicates were run, and the best fitting model in each case was selected according to the lowest BIC.

### Analysis of Spatial Biases in the Genomic Distribution of NUPTs

For every species, genomic coordinates of NUPTs were obtained from tabular BLASTN files containing the alignments between plastid and nuclear genomes. Genomic coordinates for nuclear structural genes were obtained from the original genomic feature files (gff3) for each genome (Table S1); only the longest isoforms were considered. For the remaining genomic features, gff3 files newly generated during the genome reannotation step described above were employed.

The R package *GenomicDistributions* v1.8.0 (Kupkova et al. 2022) was employed to perform the overlap analysis based on counts of overlapping bp between NUPTs and every other feature in the nuclear genome. The expected counts of overlapping bp between NUPTs and every other genomic feature were calculated using the calcExpectedPartitions function with the bpProportion argument set to TRUE to account for biases in the fraction of the genome occupied by a given feature. This function implements Pearson's Chi-squared independence tests with Yates' continuity correction to assess whether observed counts of overlapping bp between NUPTs and each genomic feature were significantly different from expected, accounting for the nonuniform distribution of genomic features. The R package *GenometriCorr* v1.1.24 (Favorov et al. 2012) was used to test whether NUPTs and every other genomic feature were located in a mutually nonrandom manner throughout the nuclear genome, i.e. showed evidence of spatial association or their locations throughout the nuclear genome were rather independent. Two different statistical tests were implemented by means of the GenometriCorr function. First, the Jaccard test employs a different approach to detect and quantify spatial overlap between NUPTs and every other genomic feature by measuring the ratio of the length (in bp) of their intersection to that of their union, i.e. the Jaccard index. Second, the RDT test assesses spatial correlation between genomic features, i.e. whether the sets of positions corresponding to two genomic features are closer together (positive spatial correlation) or farther apart (negative spatial correlation) than expected. Here, the exact distances are less important than the relative relationship, so we used the positions corresponding to the centers of each genomic feature rather than the genomic ranges themselves. Assuming the NUPTs and any other genomic feature are strictly independent, the NUPTs will be positioned randomly with respect to the genomic feature, resulting in a uniform distribution of relative distances that forms an ideal straight line. A correlation-like measure is additionally defined to determine the sign and magnitude of the deviations between the area of the distribution of observed distances and that of the expected distribution under the uniform null model. In both the Jaccard and RDT tests, to create a null distribution for comparison and report *P* values, 1,000 permutations were run by setting the permut.number to 1,000; in each permutation, the order of NUPTs was shuffled across the nuclear genome while maintaining the spacing between genomic ranges.

## Abbreviations

BIC, Bayesian Information Criterion; gff3, genomic feature file; IQR, Interquartile Range; IR, Inverted Repeat; IRE, iron responsive element; isrR, iron stress-repressed RNA; JC69, Jukes–Cantor, 69; *K*, corrected number of substitutions per site; *K*_s_, synonymous substitutions per synonymous site; Q1, first quartile; Q3, third quartile; LSC, Large Single Copy; MYA, million years ago; RDT, Relative Distance Test; snoRNA, small nucleolar RNA; SSC, Small Single Copy; TeloSII, Telo-box and Site II elements; TFBS, transcription factor binding site; TE, transposable element.

## Supplementary Material

evaf194_Supplementary_Data

## Data Availability

This study used publicly available genomes documented in the Supplementary material. Results generated from this study are included in the manuscript and Supplementary material. Gff3 genome annotation files generated in this article are available on https://doi.org/10.5281/zenodo.15639722.
