# Supplementary material for: Episodic and Ongoing Mechanisms Drive Plastid-Derived Nuclear DNA Evolution in Angiosperms
Source: Genome Biol Evol. 2025 Oct 13;17(11):evaf194. doi: 10.1093/gbe/evaf194 (PMC12572781; doi:10.1093/gbe/evaf194)
Supplement: evaf194_Supplementary_Data [file evaf194_supplementary_data.zip › Legends to Supplementary MaterialV2.docx]

**Supplementary Figures:**

**Supplementary Fig. S1. Violin plots depicting distributions of NUPTs’ ages.** *K* was used as a proxy of age. Kernel density was estimated using the Gaussian method. The corresponding boxplots are embedded**.** The mean is indicated as a blue diamond. The names of the species are abbreviated as in **Table 1**.

**Supplementary Fig. S2. Modeling the distribution of NUPTs’ ages.** *K* was used as a proxy of age. Density curves representing the distribution model selected as the best fit for each species colored according to the components of the selected model are shown. Histograms depicting the distributions of NUPTs’ *K* are also displayed. The names of the species are abbreviated as in **Table 1**.

**Supplementary Fig. S3. Correlation analysis between NUPTs’ ages and sizes.** *K* was used as a proxy of age**.** Two rank-based correlation tests were performed, *i.e*., Kendall’s and Spearman’s. The coefficients and *P-*values (*P*) from each test are displayed and the significance of the tests is indicated with asterisks. The names of the species are abbreviated as in **Table 1**.

**Supplementary Fig. S4.** **Distribution of NUPTs across the plastid genome**. The frequencies of NUPTs per bp are displayed as line-dot plots colored according to the four regions typically featuring the plastid genome, which are represented in the *x*-axis, except for Mtr, which did not show the canonical quadripartite structure. From left to right: Large Single Copy, Inverted Repeat A, Small Single Copy and Inverted Repeat B. Hotspots and coldspots of NUPTs are highlighted. The names of the species are abbreviated as in **Table 1**.

**Supplementary Fig. S5.** **Overlap analysis between NUPTs and RNA genes**. RNA genes are depicted as circles whose positions in the *y*-axis and colors represent two overlap tests, *i.e.*, counts of overlapping bp and Jaccard index, respectively. The significance of the tests is indicated as follows: (*) significant according to both, (+) only significant according to test based on counts of overlapping bp and (x) only significant according to test based on Jaccard index. From left to right: eukaryotic rRNA, prokaryotic rRNA, nuclear tRNA, plastid tRNA, mitochondrial tRNA, self-splicing intron RNA (groups I and / or II), regulatory RNA, spliceosomal RNA and other RNA. The names of the species are abbreviated as in **Table 1**.

**Supplementary Fig. S6. Overlap analysis between NUPTs and sub-classes of regulatory RNA genes.** Sub-classes of regulatory RNA genes are depicted as circles whose positions in the y-axis and colors represent two different overlap tests, *i.e.*, counts of overlapping bp and Jaccard index, respectively. The significance of the tests is indicated as follows: (*) significant according to both, (+) only significant according to test based on counts of overlapping bp and (x) only significant according to test based on Jaccard index. From left to right: microRNA, snoRNA, isrR, IRE, TeloSII-containing RNA. The names of the species are abbreviated as in **Table 1**.

**Supplementary Fig. S7. Distance analysis based on RDT tests between NUPTs and TEs**. TEs as a whole and classified in classes and orders are displayed as colored bars whose heights correspond to the measure of spatial correlation resulting from the RDT tests with respect to NUPTs, *i.e*., their tendency to be found closer (positive correlation) or farther than expected (negative correlation). Significant correlations are indicated with an asterisk. From left to right: TEs as a whole, TE class I/Retrotransposon, TE class II/DNA transposon, Retrotransposon/DIRS, Retrotransposon/LINE, Retrotransposon/LTR, Retrotransposon/PLE, Retrotransposon/SINE, DNA transposon/Crypton, DNA transposon/Helitron, DNA transposon/Maverick and DNA transposon/TIR. The names of the species are abbreviated as in **Table 1**.

**Supplementary Fig. S8. Distance analysis based on RDT tests between NUPTs and TE superfamilies.** TE superfamilies are displayed as colored bars whose heights correspond to the measure of spatial correlation resulting from the RDT tests with respect to NUPTs, *i.e*., their tendency to be found closer (positive correlation) or farther than expected (negative correlation). Significant correlations are indicated with an asterisk. From left to right: DIRS/DIRS, DIRS/Ngaro, LINE/I, LINE/Jockey, LINE/L1, LINE/R2, LINE/RTE, LTR/Bel-Pao, LTR/Copia, LTR/ERV, LTR/Gypsy, LTR/Retrovirus, PLE/Penelope, SINE/5S, SINE/7SL, SINE/tRNA, Crypton/Crypton, Helitron/Helitron, Maverick/Maverick, TIR/CACTA, TIR/hAT, TIR/Mutator, TIR/P, TIR/PiggyBac, TIR/PIF-Harbinger, TIR/Merlin, TIR/Tc1-Mariner, TIR/Transib. The names of the species are abbreviated as in **Table 1**.

**Supplementary Fig. S9.** **Distance analysis based on RDT tests between NUPTs and structural genes.** Structural genes are displayed as black bars whose heights correspond to the measure of spatial correlation resulting from the RDT tests with respect to NUPTs, *i.e*., their tendency to be found closer (positive correlation) or farther than expected (negative correlation). Significant correlations are indicated with an asterisk. The names of the species are abbreviated as in **Table 1**.

**Supplementary Fig. S10.** **Overlap analysis between NUPTs and structural genes.** Gene regions are depicted as dots whose heights and colors represent two overlap tests, *i.e.*, counts of overlapping bp and Jaccard index, respectively. The significance of the tests is indicated as follows: (*) significant according to both, (+) only significant according to test based on counts of overlapping bp and (x) only significant according to test based on Jaccard index. From left to right: promoter, exon, intron and terminator. The names of the species are abbreviated as in **Table 1**.

**Supplementary Tables:**

**Table S1. Summary of 30 angiosperm species selected for the study.** Versions of nuclear and plastid genomes used are shown as well as other information.

**Table S2. Abundance of plastid DNA in the nuclear genomes of 30 plant species.**

**Table S3. Summary of NUPT sizes.**

**Table S4. Summary of NUPT relative ages.** The corrected number of substitutions per nucleotide site, *K*, was used as proxy.

**Table S5. NUPTs contributed by each component of the exponential-Gaussian mixture model in 26 plant species.** Every NUPT was assigned according to the posterior probabilities of belonging to either one component or the other.

**Table S6. Summary of plastid genome regions corresponding to hotspots and coldspots of NUPTs.**

**Table S7. Overlap analysis between NUPTs and RNA genes.** Two overlap tests were performed, *i.e.*, counts of overlapping bp and Jaccard index, respectively; the significance of the tests at a 0.05 confidence level is indicated.

**Table S8. Overlap analysis between NUPTs and sub-classes of regulatory RNA genes.** Two overlap measures were used to perform two tests, *i.e.*, counts of overlapping bp and Jaccard index, respectively; the significance of the tests at a 0.05 confidence level is indicated.

**Table S9. Distance analysis between NUPTs and TEs categorized by class, order and superfamily.** The relative distance test (RDT) was used to measure the spatial correlation between NUPTs and TEs, *i.e*., their tendency to be found significantly closer (positive correlation) or farther than expected (negative correlation) at a 0.05 confidence level; not significant correlations are also indicated.

**Table S10.** **Spatial analysis between NUPTs and structural genes.** A distance analysis was implemented on structural genes and an overlap analysis was performed on each of their regions, *i.e.*, exons and introns, plus their promoter and terminator regions for which the 1 Kb adjacent to the first and last exons were considered, respectively. For the distance analysis the relative distance test (RDT) was used to measure the spatial correlation between NUPTs and structural genes, *i.e*., their tendency to be found significantly closer (positive correlation) or farther than expected (negative correlation) at a 0.05 confidence level; not significant correlations are indicated. For the overlap analysis two different tests were performed, *i.e.*, counts of overlapping bp and Jaccard index, respectively; the significance of the tests at a 0.05 confidence level is indicated.
