# Supplementary material for: Episodic and Ongoing Mechanisms Drive Plastid-Derived Nuclear DNA Evolution in Angiosperms
Source: Genome Biol Evol. 2025 Oct 13;17(11):evaf194. doi: 10.1093/gbe/evaf194 (PMC12572781; doi:10.1093/gbe/evaf194)
Supplement: evaf194_Supplementary_Data [file evaf194_supplementary_data.zip › Supplementary_Figures_Paper_V2.pptx]

## Slide 1
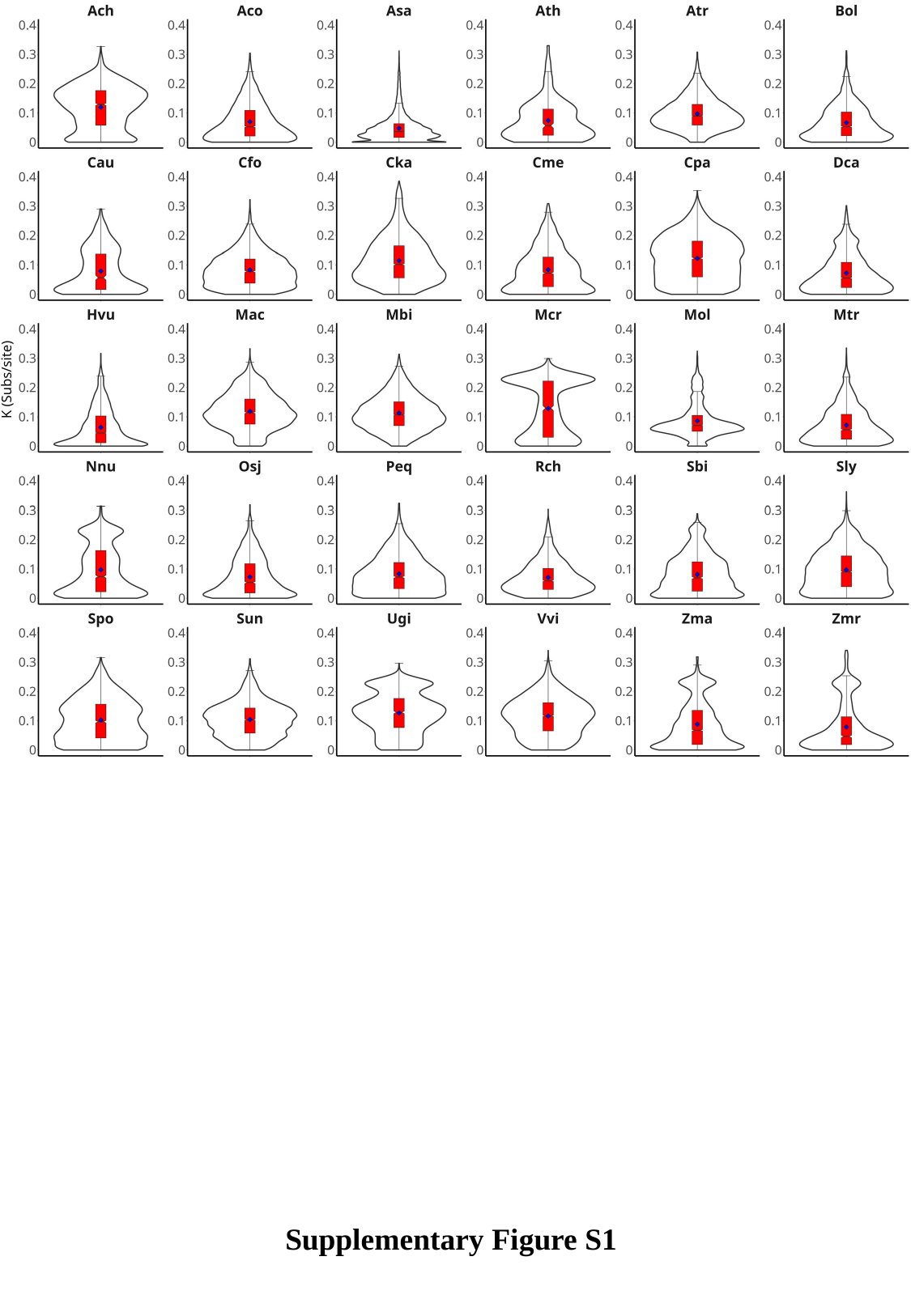

Supplementary Figure S1

## Slide 2
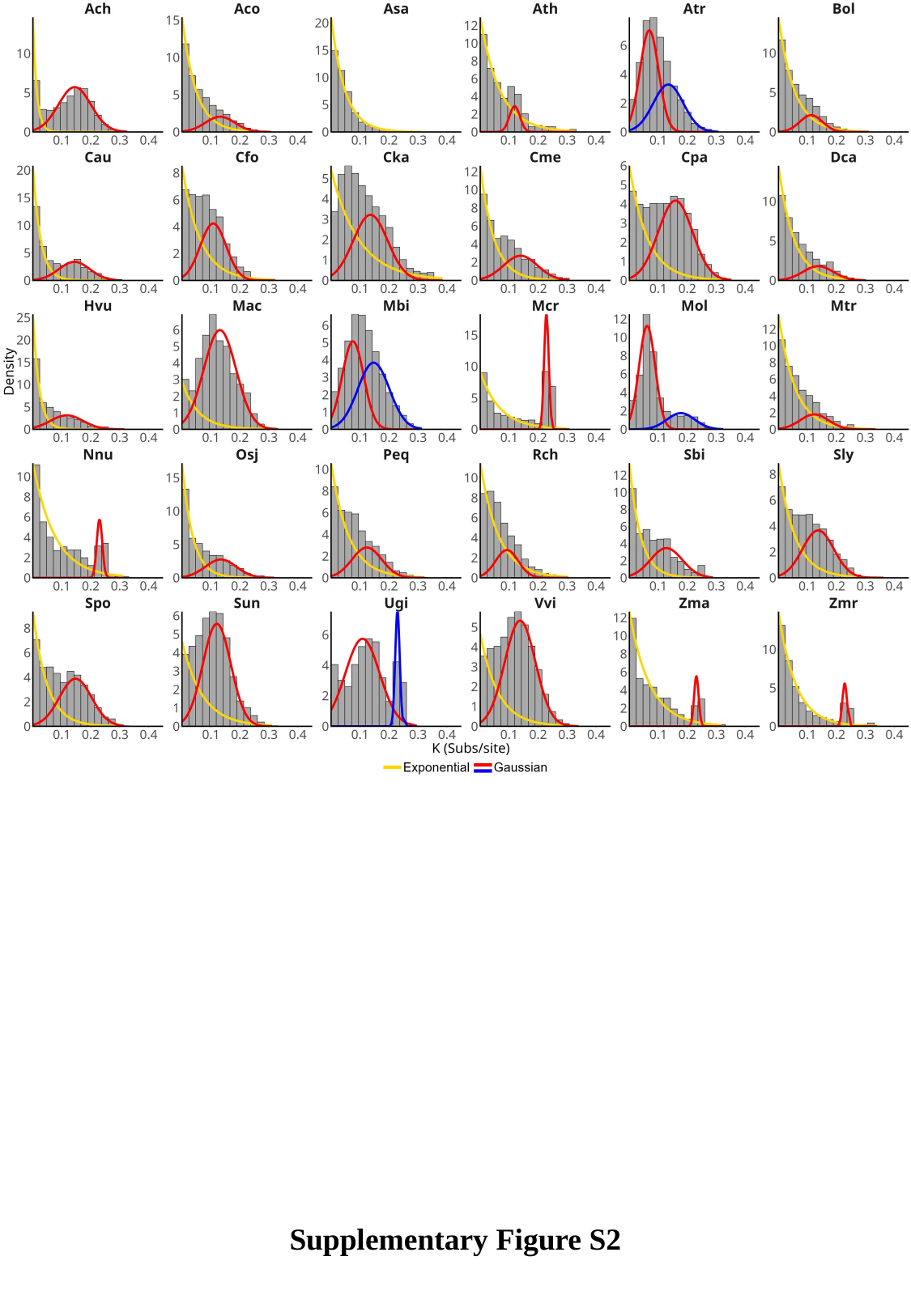

Supplementary Figure S2

## Slide 3
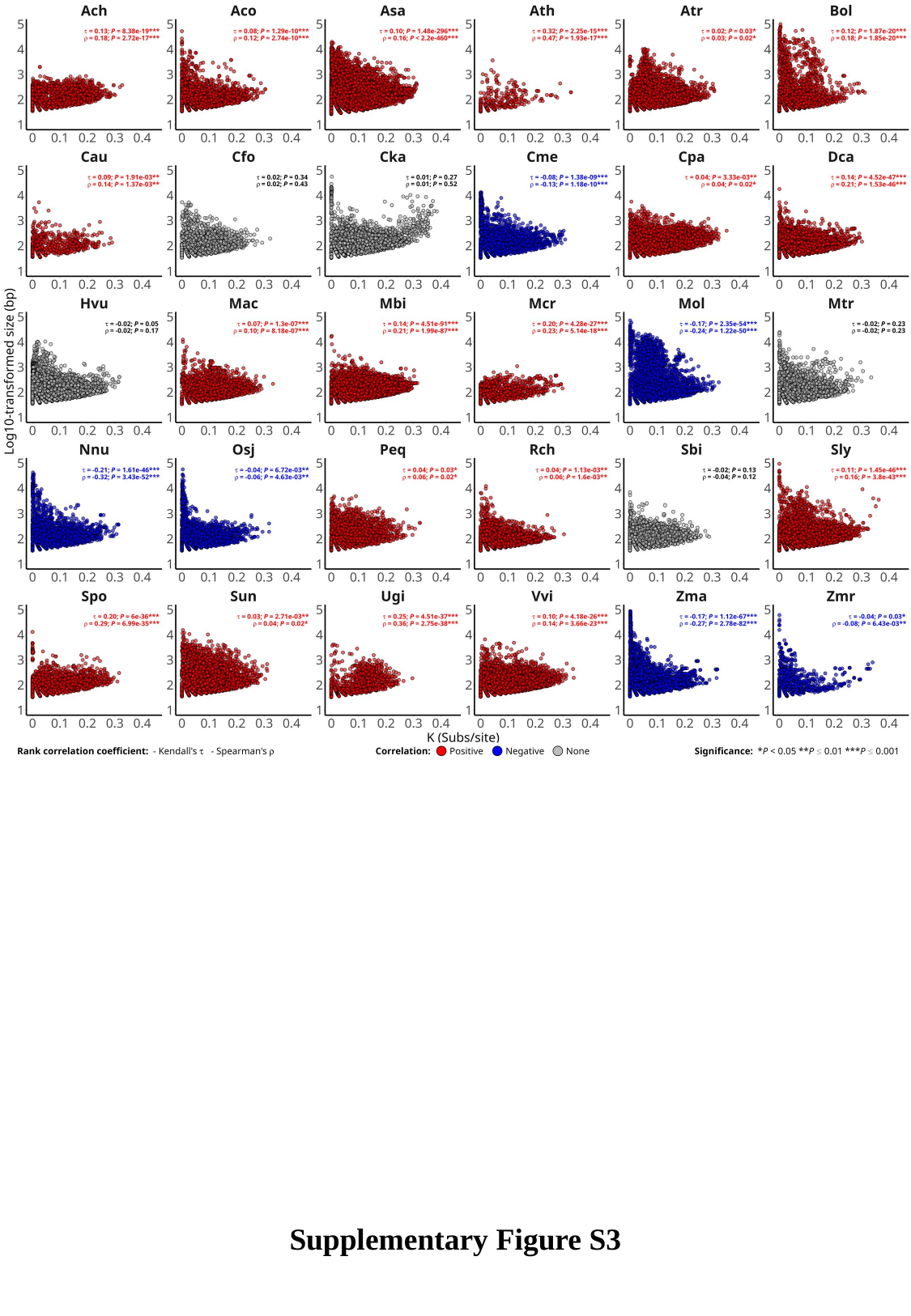

Supplementary Figure S3

## Slide 4
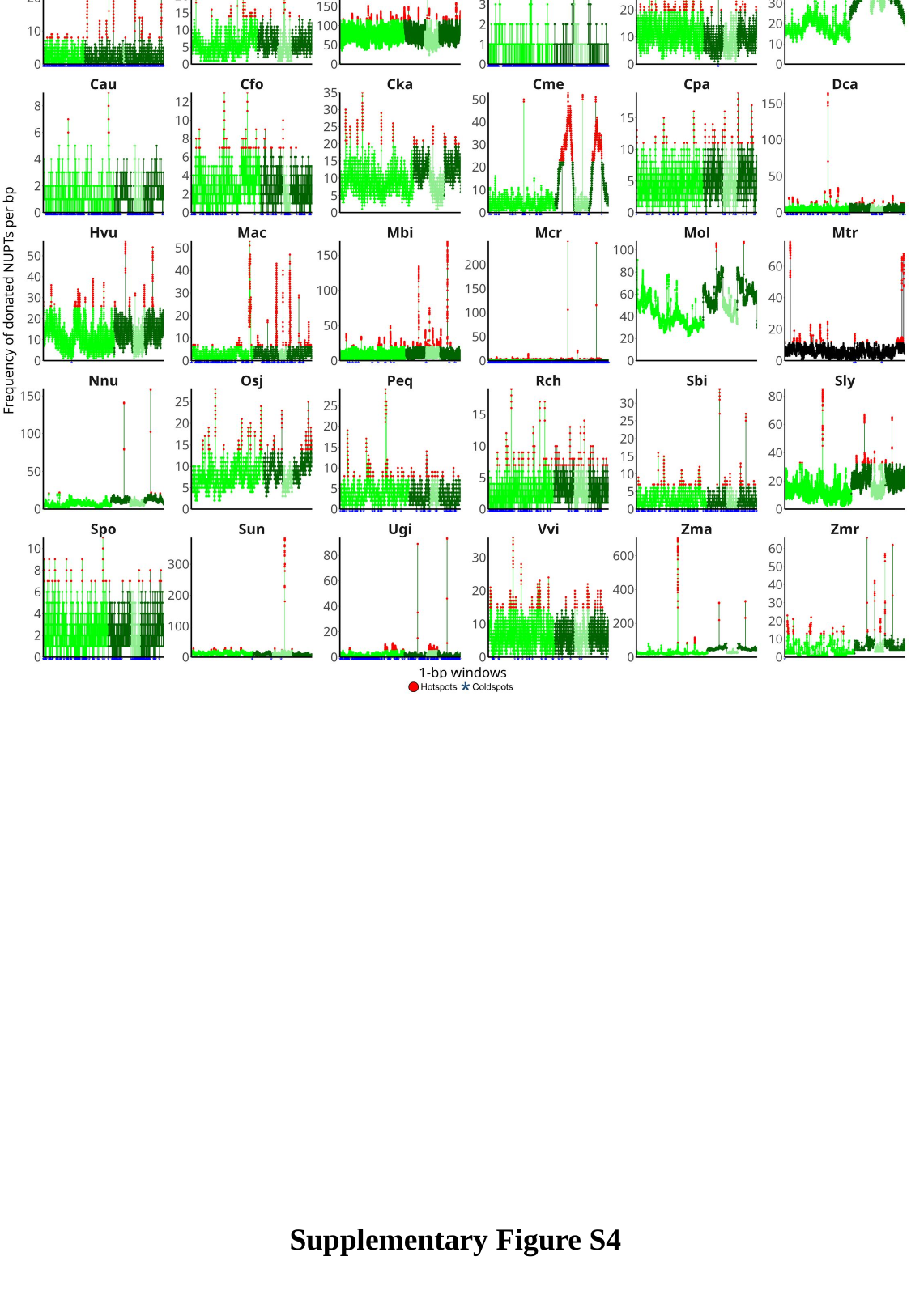

Supplementary Figure S4

## Slide 5
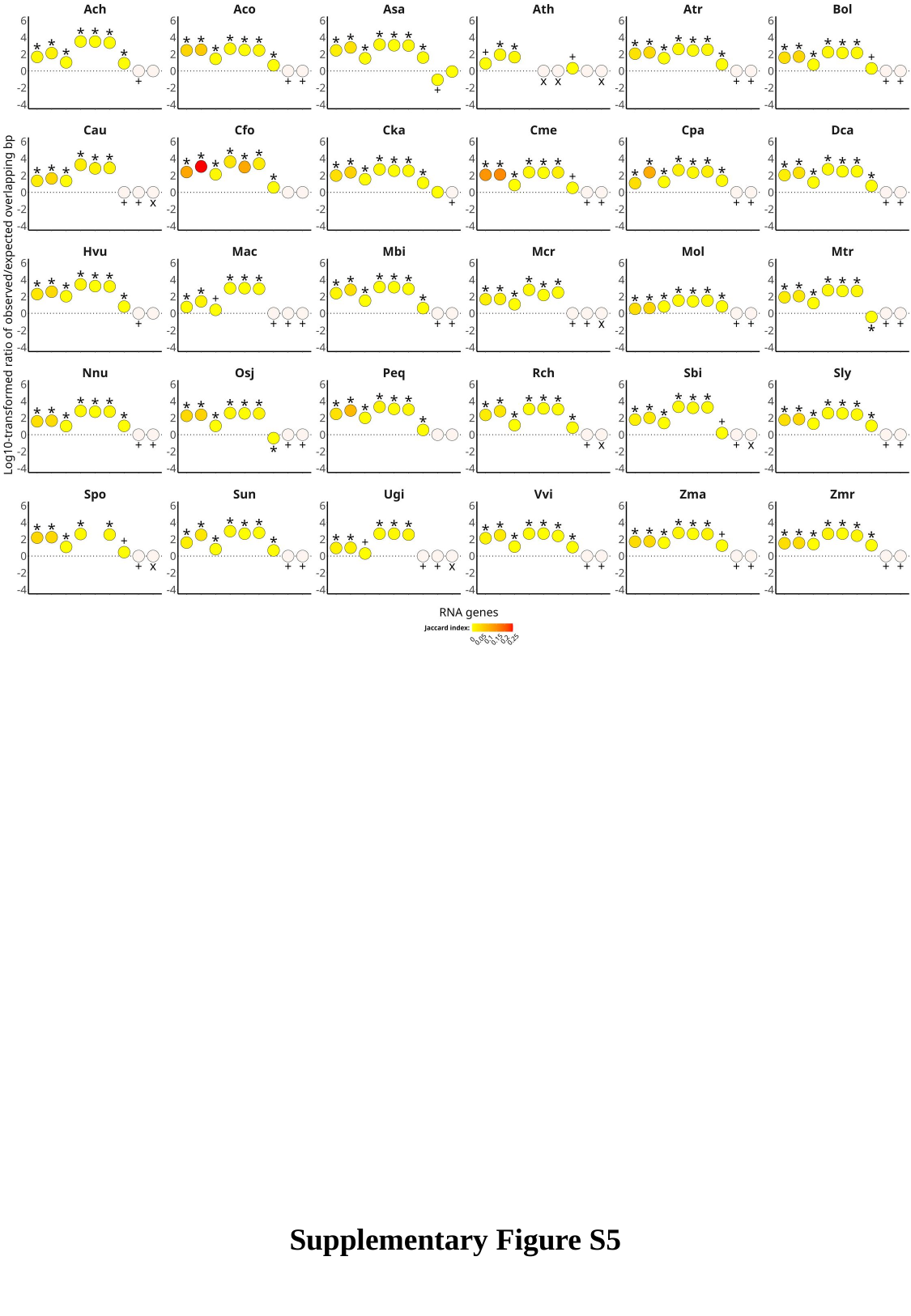

Supplementary Figure S5

## Slide 6
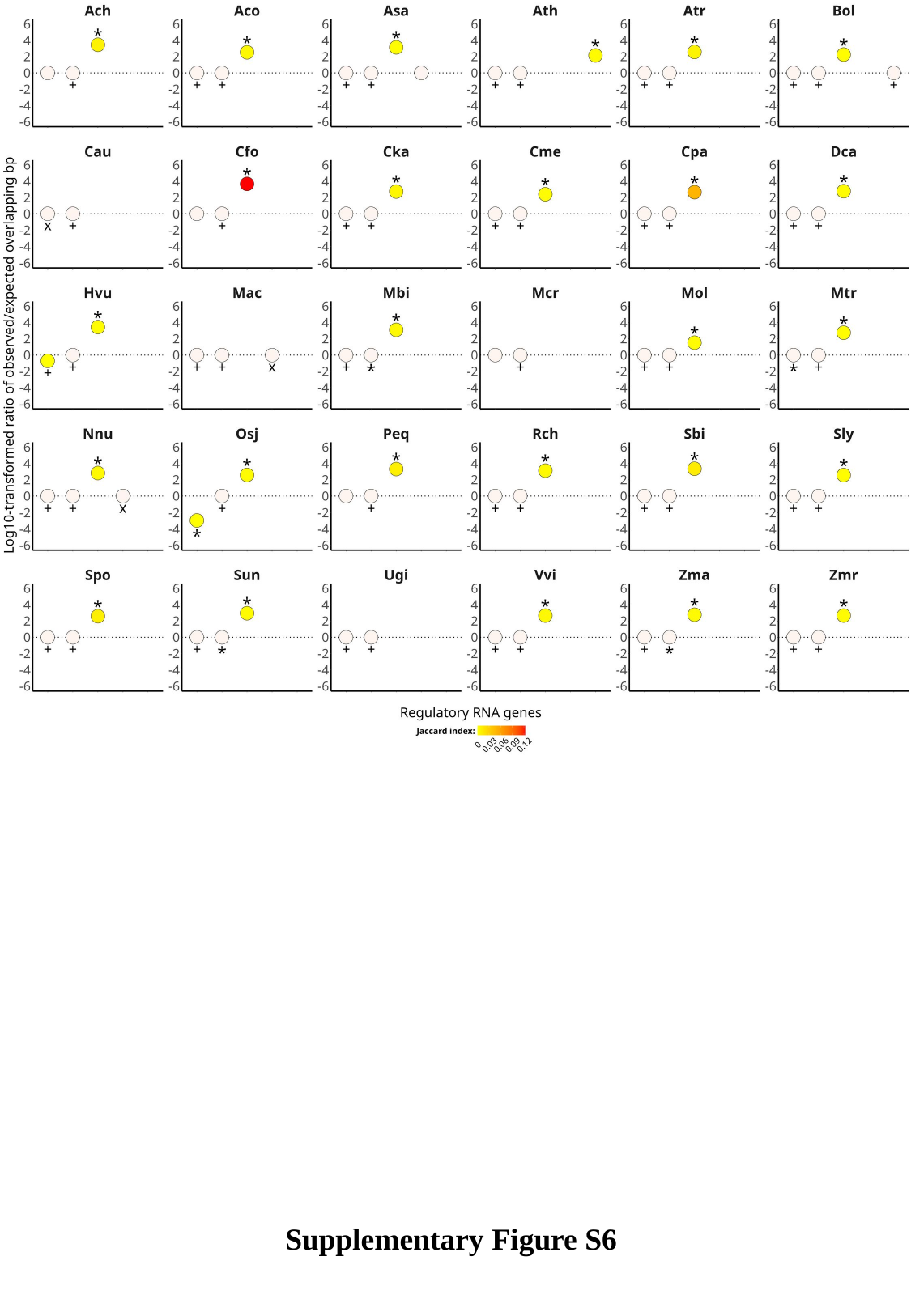

Supplementary Figure S6

## Slide 7
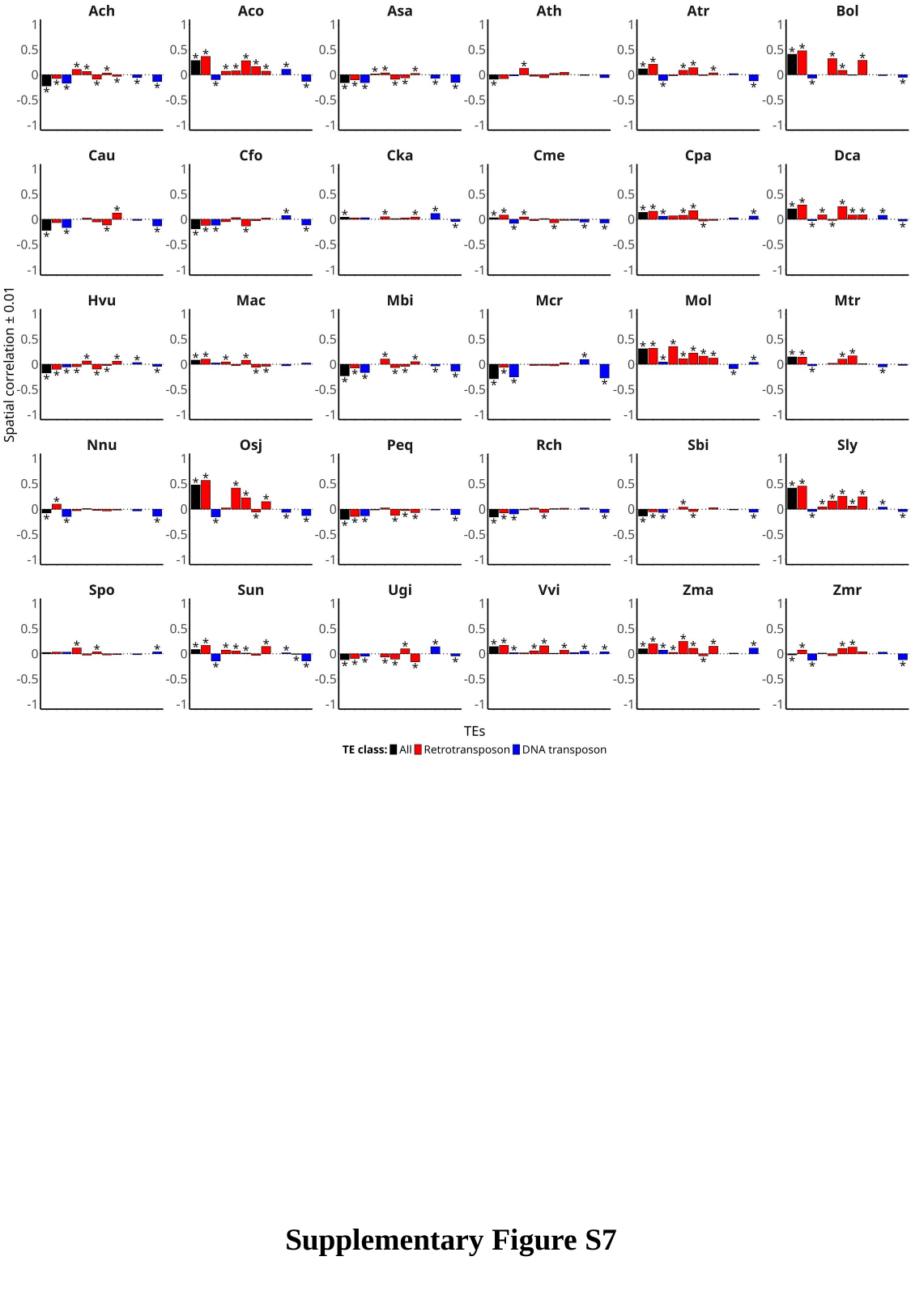

Supplementary Figure S7

## Slide 8
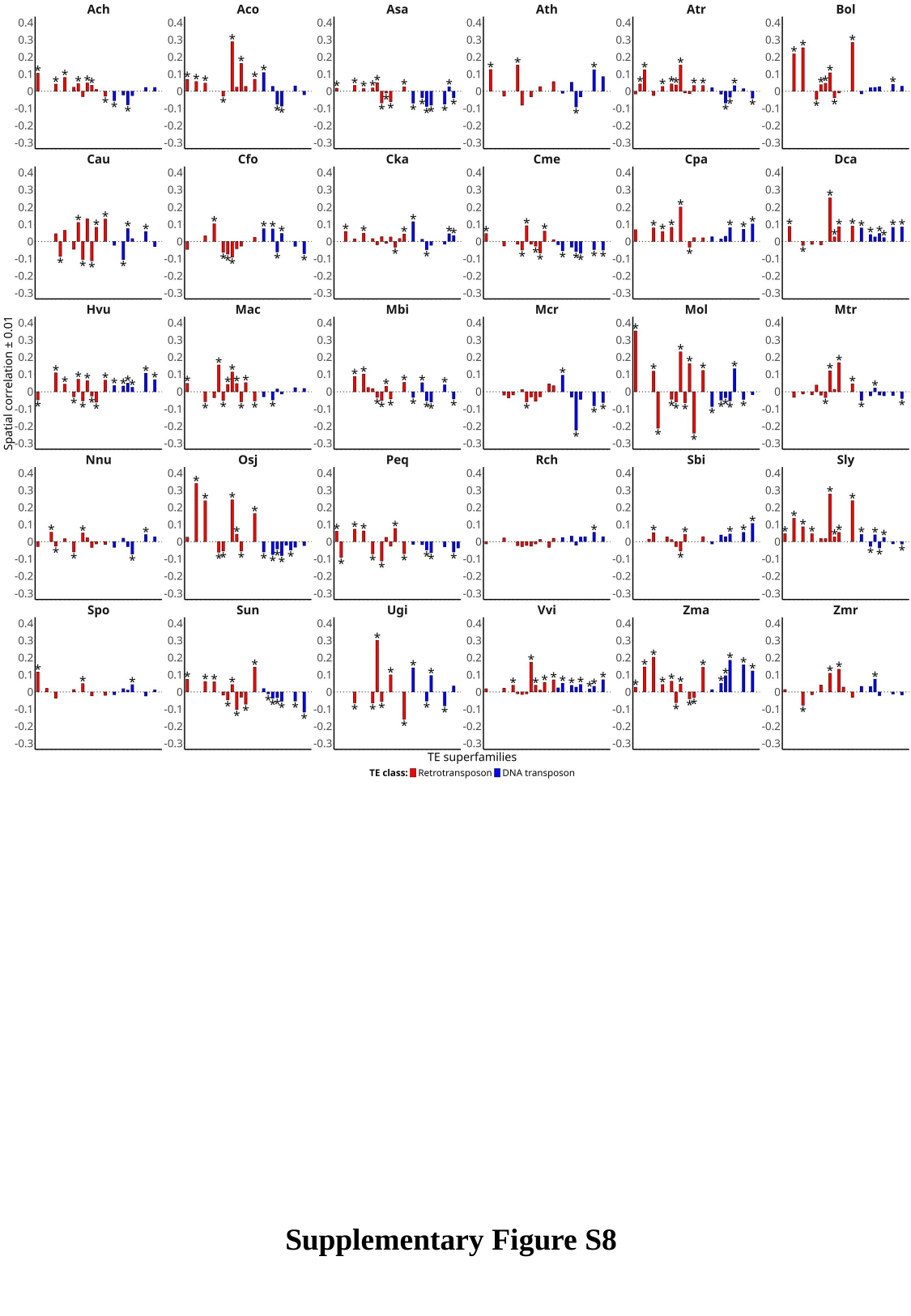

Supplementary Figure S8

## Slide 9
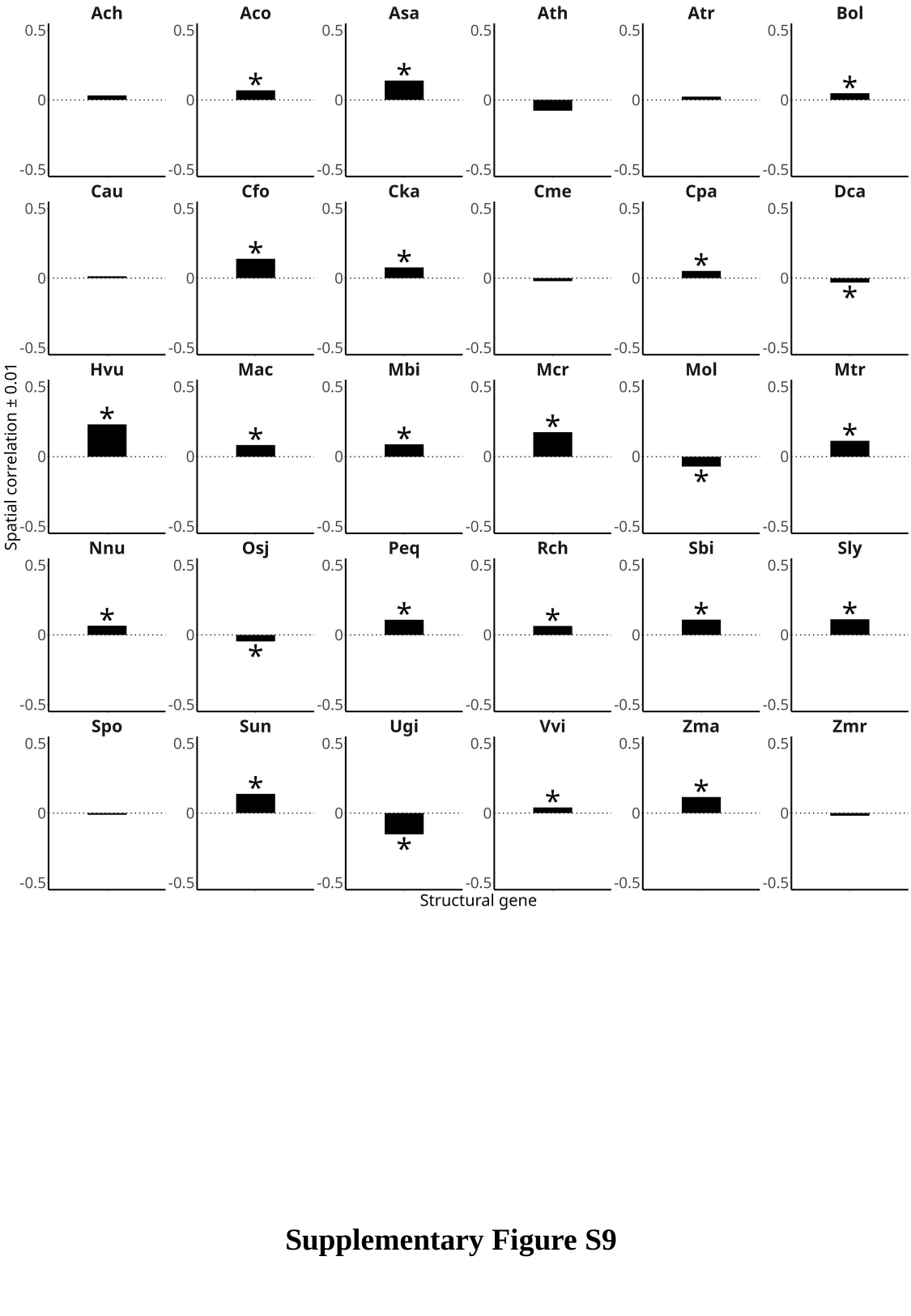

Supplementary Figure S9

## Slide 10
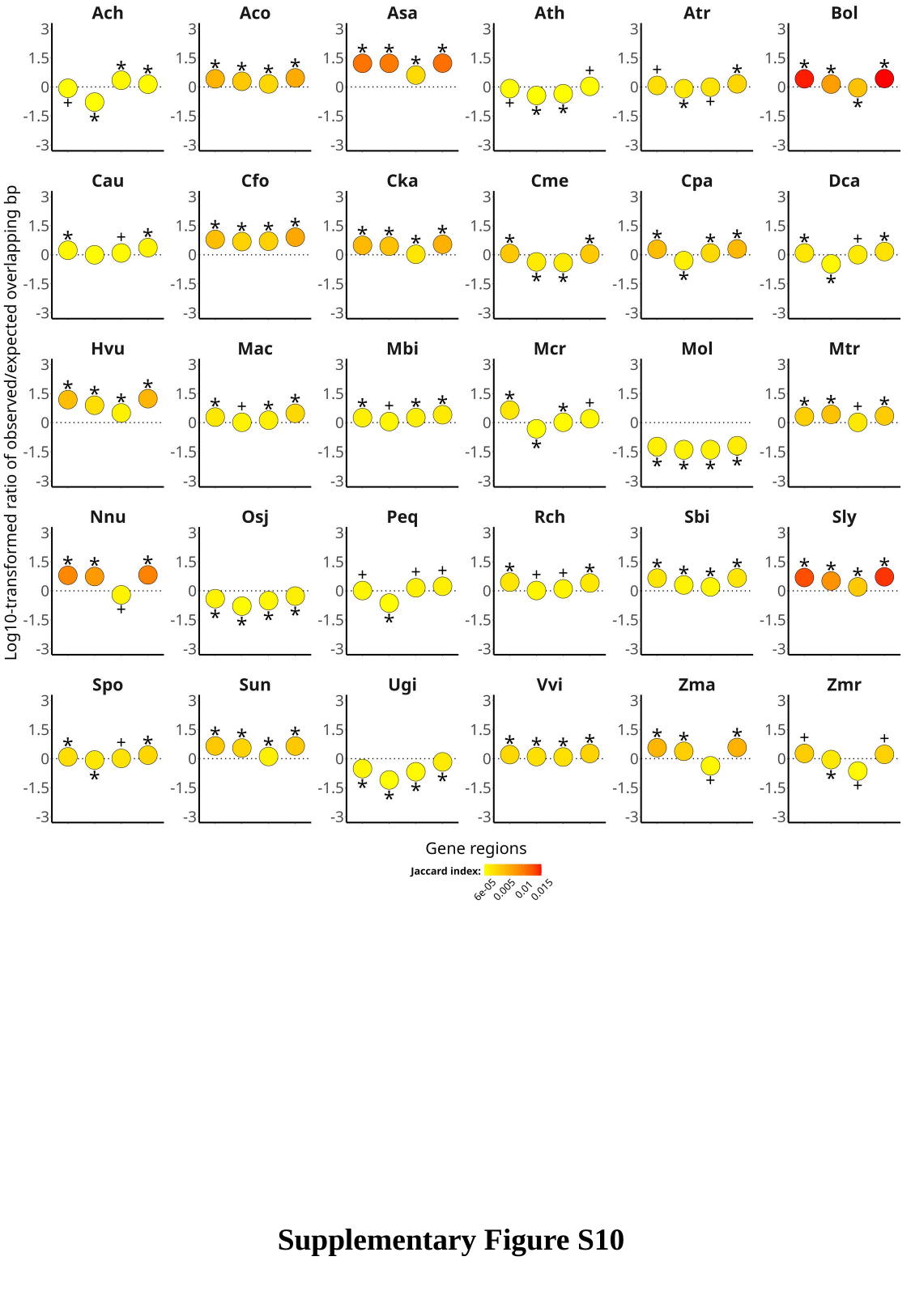

Supplementary Figure S10
